# An atypical presentation of an ovarian lymphoma: a case report

**DOI:** 10.1186/s13256-018-1884-8

**Published:** 2018-11-14

**Authors:** Chanil Deshan Ekanayake, Ramani Punchihewa, Prasantha Sudehana Wijesinghe

**Affiliations:** 1 Obstetrics and Gynaecology Unit, District General Hospital, Mannar, Sri Lanka; 2 Department of Pathology, District General Hospital, Kalutara, Sri Lanka; 3 Department of Obstetrics & Gynaecology, Faculty of Medicine, University of Kelaniya, Ragama, Sri Lanka

**Keywords:** Ovarian lymphoma, Non-Hodgkin’s lymphoma, Case report

## Abstract

**Background:**

Ovarian lymphoma has a varied clinical presentation and rarely presents with heavy menstrual bleeding. It may occur *de novo* or secondary to systemic disease and macroscopically appear as solid ovarian tumors.

**Case presentation:**

A 32-year-old Tamil woman presented with heavy menstrual bleeding of 4 months’ duration. On examination she was anemic with no lymphadenopathy. A large immobile pelvic mass and three firm nodules were found involving her vaginal walls. Ultrasonography suggested a fibroid uterus with two large pedunculated fibroids. Following preoperative optimization an endometrial sampling and biopsy of the nodules were done. Subsequently, histology revealed proliferative phase endometrium. The vaginal nodules showed lymphoid tissue.

She presented a week later with an undulating fever and features of acute abdomen with clinical evidence of ascites. During an emergency laparotomy two large solid ovarian masses, gross ascites, pelvic lymph nodes, para-aortic lymph nodes, mesenteric lymph nodes, omental deposits, and a 24-week-size uterus were found. Bilateral oophorectomy was done. Laboratory investigations revealed raised lactate dehydrogenase with normal serum β-human chorionic gonadotropin, alpha-fetoprotein, and cancer antigen-125 levels. Histology of ovarian specimens revealed a diffuse large B cell lymphoma.

A bone marrow biopsy revealed more than 80% infiltration with lymphoid cells. Two weeks after the laparotomy a computed tomography of her chest, abdomen, and pelvis revealed a pelvic mass, gross ascites, omental deposits, hepatosplenomegaly, and enlarged lymph nodes above and below her diaphragm. Immunohistochemistry confirmed the diagnosis of B cell lymphoblastic lymphoma. She was classified as stage IV E non-Hodgkin’s lymphoma on the Ann Arbor staging system.

**Conclusion:**

This is an atypical presentation of an ovarian lymphoma. The atypical presentations of ovarian lymphomas can lead to diagnostic dilemmas.

## Background

Lymphomas presenting with gynecological symptoms are rare. Involvement of the ovary by malignant lymphoma can be primary or secondary [1–3]. The difference between primary and secondary lymphomas is important in terms of prognosis [4]. Primary ovarian lymphomas present as solid ovarian tumors with an overall incidence of 0.5% of all non-Hodgkin’s lymphomas (NHLs) and 1.5% of all ovarian malignancies [5]. Secondary deposits can also occur following dissemination of systemic disease [6]. For therapeutic purposes ovarian lymphomas are regarded as manifestations of systemic disease and staged as per the Ann Arbor classification [7].

## Case presentation

A 32-year-old Tamil woman presented with heavy menstrual bleeding of 4 months’ duration. She had no previous gynecological issues and had delivered two children vaginally. Her past medical history and family history were unremarkable. There were no psychosocial stresses. On examination she was pale, had no palpable lymph nodes, no hepatosplenomegaly, a large pelvic mass, and three firm vaginal nodules. Her hemoglobin was 5.2 g/dl and she had a white blood cell count of 9100/mm^3^ with 50% lymphocytes and platelets of 487,000/mm^3^. Blood picture showed evidence of microcytic anemia. A pelvic ultrasound suggested a fibroid uterus with two large pedunculated fibroids. Following preoperative optimization, dilatation and curettage and biopsy of the vaginal nodules were done. Histology revealed proliferative phase endometrium. The vaginal nodules showed lymphoid tissue.

**Fig. 1:**
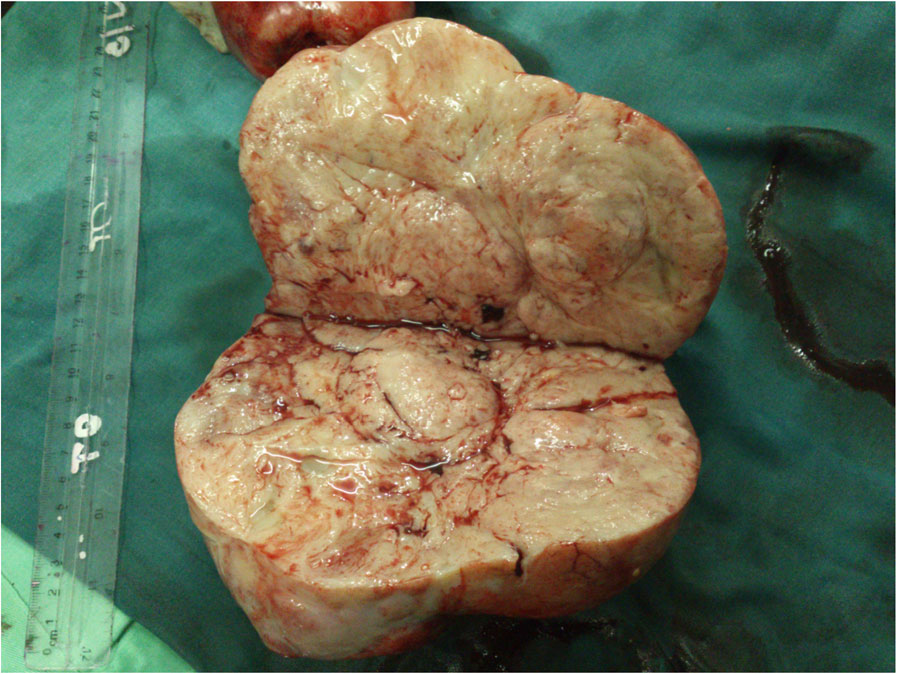
Cut surface of right ovarian tumor

A week later, she developed fever, features of an acute abdomen, and ascites. Her white blood cell count had risen to 36,000/mm^3^ with predominant lymphocytes. An emergency laparotomy was done which revealed two solid ovarian masses (Fig. 1), gross ascites, omental deposits, enlarged mesenteric lymph nodes, and a bulky uterus with thickened infundibulopelvic pedicles. A bilateral oophorectomy was done for histological diagnosis and to relieve bowel compression. A hysterectomy was not done as there was pelvic side wall involvement. Her lactate dehydrogenase (LDH) was 2250 IU/L with normal serum β-human chorionic gonadotropin (β-hCG), alpha-fetoprotein (AFP), and cancer antigen-125 (CA-125) levels. Histology revealed a diffuse large B cell lymphoma. As her general condition was deteriorating, she was started on the cyclophosphamide, adriamycin, vincristine, and prednisolone (CHOP) chemotherapy regimen while awaiting staging investigations. She had a dramatic clinical improvement with the first cycle of chemotherapy.

A bone marrow biopsy revealed 80% infiltration with lymphoid cells. Imaging showed enlarged lymph nodes above and below her diaphragm, ascites, and hepatosplenomegaly. Immunohistochemistry revealed focal CD20 staining and nuclear positivity for terminal deoxynucleotidyl transferase (TdT) and scattered CD3 positivity which suggested a B cell lymphoblastic lymphoma. Considering the blood count with bone marrow findings, a diagnosis of lymphoblastic lymphoma/leukemia was made according to World Health Organization (WHO) classification [8]. Cerebrospinal fluid (CSF) cytology was normal. Cytogenetics was not done due to financial constraints.

**Fig. 2:**
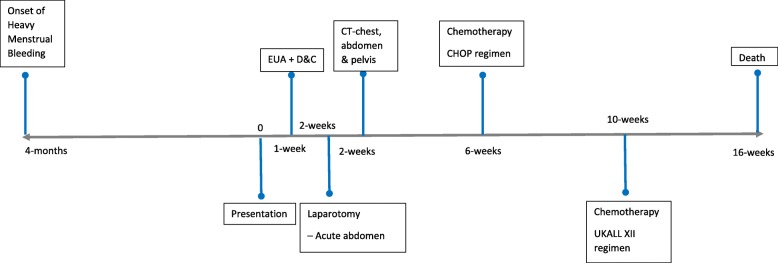
Timeline showing disease progression in patient. *CHOP* cyclophosphamide, adriamycin, vincristine, and prednisolone chemotherapy regimen, *CT* computed tomography, *D&C* dilatation and curettage, *EUA* evaluation under anesthesia, *UKALL XII* prednisolone, vincristine, daunorubicin, asparaginase and intrathecally administered methotrexate prednisolone

She was later commenced on UKALL XII Trial protocol containing prednisolone, vincristine, daunorubicin, asparaginase, and intrathecally administered methotrexate and was in remission at the end of phase 1 induction chemotherapy. However she developed hepatotoxicity which precluded continuation of chemotherapy and eventually she died. A timeline to show disease progression is shown in Fig. 2.

## Discussion

The initial blood picture in this patient suggested a microcytic anemia likely to be due to heavy menstrual bleeding. The subsequent endometrial sampling did not contribute to the eventual diagnosis. Even the presentation of an acute abdomen following endometrial sampling suggested a surgical complication rather than a hematological malignancy. Furthermore, the operative findings of ascites and solid ovarian masses can also be interpreted as a disseminated ovarian malignancy. Only the mesenteric nodes and the abnormal appearance of the uterine ligaments suggested a more sinister cause.

The common differential diagnosis of solid ovarian tumors includes Brenner tumors, teratomas, dysgerminomas, ovarian fibromas, ovarian thecomas, granulosa cell tumors, and Krukenberg tumors [9]. A definitive pathological diagnosis was made after immunostaining. In our case CD20, TdT, and scattered CD3 positivity suggested a B cell lymphoblastic lymphoma. Secondary involvement of the genital tract by NHL is a rare presentation ante-mortem. In these patients, the ovaries are predominantly involved by B cell phenotypes, of which diffuse large B cell lymphoma is the commonest subtype.

It is difficult to determine whether the lymphoma is primary or secondary once it is disseminated. Although a generalized disease with secondary ovarian deposits is commoner than a primary ovarian lymphoma, factors which suggested the latter were: an initially normal white cell count and a blood picture showing only microcytic anemia and the operative findings of the largest deposits being ovarian deposits. Whether it was a primary or secondary ovarian lymphoma was important only in terms of prognosis as all lymphomas of the ovary should be regarded as local manifestations of systemic disease for therapeutic purposes [4, 7]. Furthermore, the prognosis of ovarian lymphomas is often poor because of delayed diagnosis. The best treatment option seems to be chemotherapy and gynecologists should be aware of this rare presentation to avoid unnecessary radical surgery [10].

Unfortunately, despite an initial rapid recovery following chemotherapy, the progressive hepatic dysfunction ruled out the possibility of continuing chemotherapy which led to disease progression and the eventual death of our patient.

## Conclusions

Ovarian lymphomas will present with atypical signs and symptoms which raise diagnostic and therapeutic dilemmas. In these rare instances, they can mimic ovarian malignancy and can lead to inevitable surgical interventions.
